# Insights into Pulmonary Arterial Hypertension in Connective Tissue Diseases

**DOI:** 10.3390/jcm14134742

**Published:** 2025-07-04

**Authors:** Bogna Grygiel-Górniak, Mateusz Lucki, Przemysław Daroszewski, Ewa Lucka

**Affiliations:** 1 Department of Rheumatology, Rehabilitation and Internal Diseases, Poznan University of Medical Sciences, 61-701 Poznań, Poland; bgrygiel@ump.edu.pl; 2 Department and Clinic of Cardiology, University of Medical Sciences, 60-545 Poznań, Poland; mateusz.lucki@usk.poznan.pl; 3 Department of Organization and Management in Healthcare, Poznań University of Medical Sciences, 60-545 Poznań, Poland; dyrektor@orsk.pl; 4 Clinical Rehabilitation Laboratory, Department of Rehabilitation and Physiotherapy, University of Medical Sciences, 60-545 Poznań, Poland

**Keywords:** connective tissue diseases, pulmonary arterial hypertension, risk factors, treatment strategies

## Abstract

Pulmonary arterial hypertension (PAH) is a severe complication associated with connective tissue diseases (CTDs), which is characterized by a significant influence on the patient’s prognosis and mortality. The prevalence of PAH varies depending on the type of CTD. Still, it is highly prevalent in patients with systemic sclerosis (SSc), systemic lupus erythematosus (SLE), mixed connective tissue disease (MCTD), and primary Sjögren’s syndrome (pSS). Identifying rheumatic disease-specific risk factors is crucial for early diagnosis and intervention. Risk factors for PAH development include specific sociological factors (related to race, gender, and age), clinical features (particularly severe Raynaud’s phenomenon and multiple telangiectasias), cardiological factors (pericarditis and left heart disease), biochemical factors (elevated NT-proBNP and decreased HDL-cholesterol), serological factors (presence of ANA, e.g., anti-U1-RNP or SSA, and antiphospholipid antibodies), and pulmonary factors (interstitial lung disease and decreased DLCO or DLCO/alveolar volume ratio < 70%, FVC/DLCO > 1.6). The analysis of risk factors can be the most useful during the selection of patients at high risk of PAH development. The initial diagnosis of PAH is usually based on transthoracic echocardiography (TTE) and is finally confirmed by right heart catheterization (RHC). Targeted therapies can improve outcomes and include endothelin receptor antagonists, prostacyclin analogs, phosphodiesterase inhibitors, and tailored immunosuppressive treatments. Effective management strategies require a multidisciplinary approach involving rheumatologists, cardiologists, and pulmonologists. The risk stratification and individualized treatment strategies can enhance survival and quality of life in patients with PAH-CTD.

## 1. Introduction

PAH appears more often than clinicians usually suspect. The development of this complication is associated with the need to quickly solve many clinical problems in everyday cardiology or rheumatology practice. Since the prevalence of pulmonary arterial hypertension in CTD varies between different diseases (PAH is the most prevalent in SSc or SLE), populations (e.g., increased PAH-SSc incidences in European countries, and PAH-SLE in Asian regions), and races (e.g., highly prevalent PAH-SSc in African Americans) we decided to describe the PAH risk factors that are common and independent of ancestry. Unfortunately, the diagnosis of PAH is delayed due to many non-specific symptoms and an insidious onset of pulmonary pathological changes, causing a delay in the treatment. Therefore, knowing the risk factors related to PAH development may help in fast and efficient diagnostics. These risk factors depend on specific features of diseases, e.g., in SSc, the risk is higher if patients present severe Raynaud’s phenomenon, severe fingertip ulcers [1], and many telangiectasias on physical examination [2]. The risk factor increasing PAH prevalence in pSS is the presence of anti-SSA/Ro antibodies in serum [3]. Some data emphasize the correlation of PAH with high SLE activity related to pericardial effusion, mild ILD, and increased serological activity [4,5,6,7,8,9,10,11,12].

Proper cardiological diagnosis is possible due to the implementation of appropriate diagnostic procedures. Traditionally, transthoracic echocardiography (TTE) is a noninvasive and inexpensive diagnostic tool for predicting pulmonary hypertension. Although it is commonly used for the initial diagnosis of PAH, right ventricular catheterization is the gold standard diagnostic [13]. The choice of diagnostic method drastically affects the detection of PAH. For example, the incidence of PAH in SSc measured by right ventricular catheterization is 8%, while that detected by screening echocardiogram is almost 27% [14]. The implementation of adequate diagnostic procedures in patients with many risk factors for PAH not only leads to early detection of PAH but also to faster treatment and lower mortality related to increased PAH development. On the other hand, such a procedure prevents unnecessary therapy in patients with a low risk of PAH. Therefore, in the case of significant suspicion of PAH on ECHO, it should be confirmed by right ventricular catheterization, which is currently the standard diagnostic procedure.

In this review, we intend to analyze the risk factors of PAH development in the course of CTDs and discuss the current diagnosis and treatment standards of PAH crucial in cardiological and rheumatological practice. Treatment standards in our article are based on ESC 2022 guidelines for the diagnosis and treatment of pulmonary hypertension and were developed by the authors to summarize and illustrate key therapeutic steps [15]. Such knowledge may help clinicians to identify and screen patients with the highest risk of PAH development, enabling them to maximally shorten the time to the PAH diagnosis and the quick therapy implementation.

## 2. Review Methodology

This review explores the prevalence and treatment strategies for pulmonary arterial hypertension (PAH) in the context of connective tissue diseases (CTDs). The review synthesizes key findings related to epidemiology and therapeutic strategies, including the role of targeted vasodilator therapies and immunosuppressive regimens, while also highlighting the impact of multidisciplinary care and risk-stratification tools on treatment outcomes. A research question was formulated based on the PICO framework: In patients with connective tissue diseases (P), what are the risk factors and characteristics of targeted pulmonary arterial hypertension treatment (I) compared to standard management (C), with particular emphasis on diagnostic findings and description of therapeutic strategies (O)?

A literature search was conducted using the PUBMED, Medline, Web of Science, Scopus, and DOAJ databases, covering publications from January 2009 to December 2024. The search terms included ‘pulmonary arterial hypertension’ OR ‘PAH’ AND ‘connective tissue diseases’ OR ‘CTD’. Only peer-reviewed articles in English were included, focusing primarily on original studies and comprehensive reviews and current clinical practice guidelines, particularly from the European Society of Cardiology (ESC) that addressed epidemiological patterns and therapeutic approaches for PAH in CTD populations.

The analysis excluded articles based on animal models, in vitro experiments, case reports, editorial comments, and conference abstracts. Titles and abstracts were initially screened to identify relevant literature, followed by a full-text assessment of selected studies. Special emphasis was placed on data regarding PAH prevalence in systemic sclerosis (SSc), systemic lupus erythematosus (SLE), mixed connective tissue disease (MCTD), undifferentiated connective tissue disease (UCTD), rheumatoid arthritis (RA), primary Sjögren’s syndrome (pSS), and CTDs associated with vasculitis.

**Figure 1:**
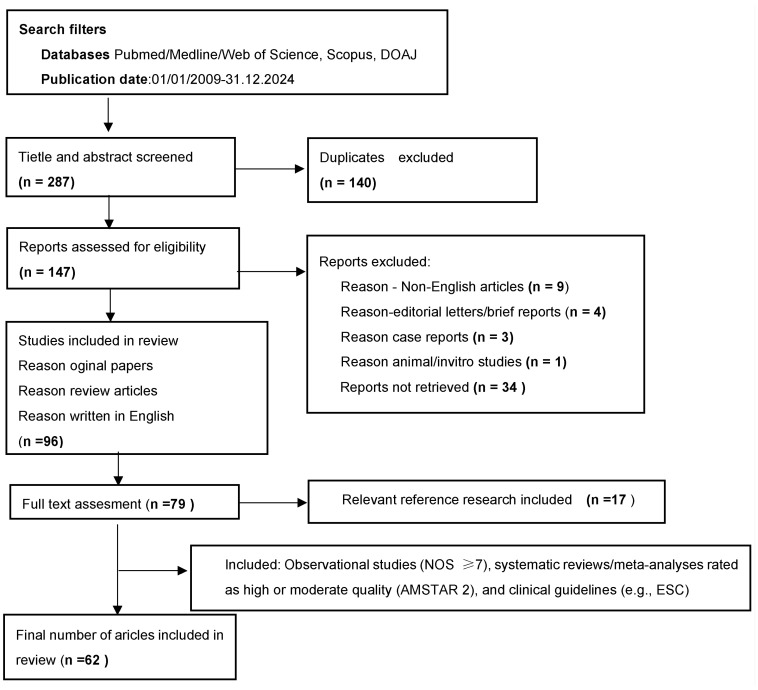
Process of screening and selection of publications included in the review.

The methodological quality of the included studies was assessed using validated appraisal tools. Observational studies were evaluated using the Newcastle-Ottawa Scale (NOS), with only studies scoring at least 7 out of 9 points being included. Only systematic reviews and meta-analyses rated as high or moderate quality using the AMSTAR 2 tool were considered. Clinical practice guidelines, such as those issued by the European Society of Cardiology, were not subject to methodological scoring but were included due to their recognized authority and relevance in clinical decision-making. Additional relevant publications were identified through citation tracking and reference review of selected articles. The selection process of publications included in the review is illustrated in Figure 1.

## 3. PAH Prevalence

PAH prevalence depends on the analyzed population. For example, in Caucasians, the most common cause of PAH is systemic sclerosis. This is reflected by the data from the large European pulmonary hypertension registry COMPERA, which describes the survival in patients with newly diagnosed PAH associated with SSc, SLE, MCTD, UCTD, RA, Sjogren syndrome, vasculitis, and other types of various connective tissue diseases (CTDs). It shows that the highest mortality is in patients with SSc-PAH compared to other CTDs associated with PAH [16]. According to the American College of Cardiology (ACC) and the American Heart Association (AHA), PAH develops in 8% of SSc patients [14] and a similar prevalence is reported in Europe (9%) [17]. In the Asian population, PAH is much more common in SLE than SSc, and the prevalence of SLE-PAH varies from 2.8% to 49% of patients (Chinese data) [5,18,19]. SLE is the most common CTD-related disease with PAH in the Chinese population, more common than SSc [5]. PAH is also considered to develop more frequently in the African American population compared to those of European ancestry [6]. The prevalence of PAH in specific CTDs depends on the analyzed populations. In Japan, MCTD is the leading cause among CTDs related to PAH, accounting for 43%, followed by SLE (29%), in contrast to PAH-CTD patients in the USA and Europe, where SSc is the leading cause of PAH [20].

### 3.1. Risk Factors of PAH in Systemic Sclerosis

The frequency of PAH is similar in lcSSc and dcSSc [21]. However, an increased risk of developing PAH is seen in patients with severe peripheral vascular disease, characterized by more severe Raynaud’s phenomenon and more extended fingertip ulcers [1], and in patients with more telangiectasias on physical examination [2]. Analysis of the PHAROS (the Pulmonary Hypertension Assessment and Recognition of Outcomes in Scleroderma Registry) population shows that race (African American) and gender (male) are associated with an increased risk of SSc-PAH [22,23].

The risk of PAH increases with age and longer disease duration. Furthermore, late-onset SSc increases the risk of PAH [17,24]. The study of Kolton Schneider et al. documented a 22% increase in the risk of developing PAH every 10 years from the moment of disease onset [25]. A nucleolar pattern of ANA and the presence of antibodies against centromere antibodies (ACA), Th/To, anti-beta2-glycoprotein U1-ribonucleoprotein, and the absence of anti-Scl 70 antibodies are associated with a higher risk of SSc-PAH.

Isolated decrease in diffusing capacity of carbon monoxide (DLCO) is an excellent predictor of subsequent development of isolated PAH in lcSSc and predicts the occurrence of PAH after 10 years of SSc [1]. Moreover, a decreased DLCO/alveolar volume ratio below 70% and increased N-terminal pro-brain natriuretic peptide (NT-proBNP > 97th percentile of normal) are also risk factors for SSc-PAH [26]. Furthermore, the ratio of forced vital capacity (FVC)/DLCO above 1.6 and increasing right ventricular systolic pressure (RVSP) > 2 mmHg/year augments the development of SSc-PAH [4,27].

### 3.2. Risk Factors of PAH in Systemic Lupus Erythematosus

Many biomarkers of pulmonary hypertension in SLE have been described in the literature. Specific symptoms such as serositis (mainly pericarditis, pleuritis) [4,9,10], mild ILD [9,10], and Raynaud’s phenomenon [4] increase the risk of PAH. In addition to symptoms, increased serological activity [5] is related to the higher pressure in pulmonary arteries. Recent data emphasize the role of anticardiolipin antibodies (aCL) and anti-U1-RNP [4,10], lupus anticoagulant (LA), aβ2-GPI [7], anti-Ro, anti-La [6,10], and anti–Scl–70 detected in SLE patients [11] in this process.

A positive result for U1-RNP antibody is independently associated with severe PAH and more active disease [12]. Interestingly, some studies also emphasize the role of hyperuricemia in the development of PAH, and a uric acid level ≥ 6.5 mg/dL [8] or a higher UA ≥ 7 mg/dL [12] is considered a risk factor for the development of PAH in patients with lupus. Pulmonary artery systolic pressure (PASP) significantly correlates with the Raynaud phenomenon but does not correlate with the disease duration [4]. Thus, some authors consider Raynaud’s phenomenon a marker for the severity of PAH [28].

In a Chinese population (*n* = 84 patients with PAH-SLE), Raynaud’s phenomenon, digital vasculitis, pericardial effusion, interstitial lung changes, positive anti-U1-RNP antibodies, and positive aCL (immunoglobulin G class) were associated with a significantly higher risk of PAH-SLE [12]. The strong association of lupus anticoagulant and APS with PAH suggests that thrombosis may play an important role in pulmonary hypertension in SLE patients [29].

### 3.3. Risk Factors of PAH in Mixed Connective Tissue Diseases

Pericarditis, ILD, thrombocytopenia, anti-Sm autoantibody reactivity, and absence of polyarthritis are independent predictors associated with PAH in MCTD [30]. In contrast, a meta-analysis by Hassan et al. shows that the prevalence of PAH in patients with MCTD is 12.53% [95% CI 8.30–18.48%], and no association between PAH and female gender and age is found [31]. A prospective study of 280 Hungarian patients with MCTD showed that the leading cause of 22 deaths was PAH (*n* = 9 patients), thrombotic thrombocytopenic purpura (*n* = 3), infections (*n* = 3), and cardiovascular events (*n* = 7). Death is more frequent in cases diagnosed at a younger age with MCTD, but there is no difference in disease duration (*p* = 0.835). Moreover, cardiovascular events, esophageal dysmotility, serositis, secondary antiphospholipid syndrome, and malignancy are significantly higher in deceased MCTD patients. Furthermore, the presence of anticardiolipin antibodies, anti-β2-glycoprotein antibodies, and anti-endothelial cell antibodies increases the risk of death [32].

### 3.4. Risk Factors of PAH in Rheumatoid Arthritis

Lung involvement in RA is observed in patients with genetic susceptibility with coexisting inducing factors, e.g., exposure to environmental dust or cigarette smoking. The synthesis of autoantibodies directed against lung mucosal surfaces induces chronic inflammation and activates neutrophils, which form neutrophil extracellular traps (NETs) [33]. Fayed et al. noted that postcapillary PAH occurs most frequently in patients with RA, whereas PAH associated with lung disease is commonly seen in patients with IIM and sarcoidosis [34]. The differential diagnosis of pulmonary hypertension in RA should include not only PAH but also lung disease, left ventricular failure, and venous thromboembolism [35].

Data from Canada show that RA-PAH patients are older at disease onset (64.0 vs. 53.7 years) and have lower mean pulmonary artery pressure (mPAP—41 vs. 50 mm Hg, *p* = 0.02) than patients with idiopathic PAH (iPAH); however, survival of both groups of patients is comparable [36]. Data from India show a positive correlation of PAP with disease duration and disease activity (r = 0.627 and 0.511, respectively) in a group of 75 RA patients [37]. PAP significantly correlates with RA duration and disease activity score. Interestingly, RA patients with mild pulmonary hypertension on echocardiography had lower high-density lipoprotein cholesterol (C-HDL) levels compared with RA patients with normal right ventricular systolic pressure (RVSP) [38]. Thus, low HDL levels have been suggested as a potential prognostic factor for PAH in RA patients.

Although lung disease is more frequently diagnosed in men, the female gender is a risk factor for PAH in RA [39,40]. When RA coexists with lung disease, it worsens the prognosis of RA-PAH [41]. The prognosis of patients with PAH-CTD can be calculated using death risk calculators such as the REVEAL 2.0 score (13 variables) or REVEAL Lite (6 parameters) [42].

### 3.5. Risk Factors of PAH in Primary Sjögren’s Syndrome

PAH is the leading cause of death in primary Sjögren’s syndrome. Other risk factors for death include liver damage and ILD [43]. In the study by Sato et al., patients with pSS had increased levels of anti-SSA/Ro antibodies and mildly positive antinuclear antibodies (ANA) 1:40 at the time of PAH diagnosis. Therefore, the author suggested that ANA titer and the presence of anti-SSA/Ro antibodies may increase the risk of pSS-PAH and should be added to the screening tests for PAH-pSS [3].

### 3.6. Risk Factors of PAH in Vasculitis

Mechanical obstruction of pulmonary vessels and increased pulmonary vessel wall stiffness are major risk factors for early death in Takayasu arteritis [44]. Patients with BD and pulmonary vascular changes are at increased risk of developing PAH [45]. The PAH in AAV is often associated with left heart disease and a poor prognosis. Other risk factors include chronic lung disease, left heart disease, older age, male sex, smoking history, and kidney involvement [46] (Table 1).

## 4. Pathogenesis of PAH Development in CTD

### 4.1. Pathogenesis of PAH-SSc

In SSc, PAH occurs mainly in the limited form of the disease [48] and is associated with endothelial cell proliferation and extracellular matrix deposition, which lead to thickening of the intima of capillaries and pulmonary arterioles [49]. Despite less severe pulmonary hemodynamics, survival of patients with SSc-PAH is worse than in idiopathic PAH [50]. The reason is probably the underlying pulmonary arterial vasculopathy and the reduced ability of the right ventricle (RV) to compensate for the increased afterload (PAH leads to increased RV afterload with subsequent RV dysfunction, failure, and premature death) [51,52].

Many factors are involved in the development of SSc-PAH; among them, the role of genetic factors is emphasized. The study of Tu et al. has shown that IL-7R, LCK, and HDAC1 are key genes related to immune regulation in SSc-PAH and are involved in T-cell immune regulation [53]. In SSc, T lymphocytes are recruited to tissues (e.g., lung, skin, etc.) and stimulate the secretion of cytokines that ultimately promote cell activation by macrophages, fibroblasts, and myofibroblasts. These processes precede endothelial cell dysfunction and subsequent induction of fibroblast proliferation and collagen synthesis [54].

Recently, many studies have highlighted the role of dendritic cells in the development of PAH. These cells, upon activation, increase the synthesis of inflammatory cytokines and chemokines, which in turn leads to pulmonary vascular remodeling and increased pulmonary vascular resistance. For example, circulating conventional type 2 DCs (cDCs) exhibit increased production of IL-6, IL-10, and tumor necrosis factor-α (TNF-α) following stimulation with TLR2 and TLR4 in SSc patients [55,56]. Since the activated non-classical monocytes and myeloid dendritic cells can produce enhanced levels of proinflammatory cytokines in SSc, their role in lung fibrosis is crucial [55].

Another pathogenic marker of SSc-PAH is IL-6, which is considered a biomarker of PAH in limited SSc. IL-6 levels are higher in SSc patients with PAH ≥ 5 years or with severe complications compared with patients with short duration of SSc or uncomplicated SSc [57,58]. In the early stages of SSc, IL-6 amplifies and maintains local and systemic inflammation and stimulates fibroblast transformation. This cytokine leads to perivascular infiltration of inflammatory cells, thus inducing the development of SSc-PAH [49] and is considered a marker of PAH [57]. Therefore, biologic drugs such as tocilizumab (IL-6 inhibitor) not only reduce inflammation but also reduce fibrosis [59].

In the process of SSc-PAH development, the role of one of the commonly determined cardiovascular markers, N-terminal pro-brain natriuretic peptide (NT-proBNP), is also important. It is a marker of neurohormonal activation useful in the diagnosis and prognosis of PAH. NT-proBNP is significantly higher in PAH-SSc compared to idiopathic PAH, despite less severe hemodynamic disorders. Its high values are also a predictor of shorter survival of patients [60].

Another marker that plays an important role is P-selectin glycoprotein ligand 1 (PSGL-1), which is a dimeric, mucin-like glycoprotein expressed on the surface of leukocytes and platelets. It is an adhesion molecule involved in immune cell trafficking that plays a key role in leukocyte recruitment during inflammation [61]. Mice deficient in P-selectin glycoprotein ligand 1 (PSGL-1) develop a spontaneous SSc-like syndrome. Furthermore, the lack of PSGL-1 induces a reduction in Treg cells, nitric oxide production, and estrogen receptor α expression and increases angiotensin II in the lungs of female mice, which promotes the development of PAH [62].

### 4.2. Pathogenesis of SLE-PAH

Among the various complications of SLE, PAH is a life-threatening and devastating complication. Unfortunately, its pathogenesis remains incompletely understood. Various studies have emphasized genetic susceptibility, endothelial dysfunction, chronic inflammation, and fibrotic remodeling in the pathogenesis of PAH development [63]. In SLE patients, fibrosis is generally rare and mild and similar to primary pulmonary hypertension [64]. Since ILD often coexists with PAH, a major role is attributed to prolonged hypoxemia, which is a characteristic consequence of ILD. ILD in SLE increases the risk of developing PAH, which is due to the shared mechanisms such as oxidative stress, endothelin dysregulation, and TGF-β1 activation [9].

The risk of developing PAH in SLE is also associated with elevated levels of C-reactive protein (CRP), which is considered a potential risk marker for SLE-PAH. In addition to CRP, age, anti-dsDNA antibodies, pericarditis, and SLE disease activity index (SLEDAI) are also associated with a higher incidence of ILD-PAH in patients with SLE [65].

Recent studies have emphasized the role of immunoglobulins and complement in the pathogenesis of PAH-SLE, which accumulate on the arterial walls, stimulating pulmonary vasculitis and thus contributing to the development of PAH-SLE [66,67]. Interestingly, in PAH, immune complexes preferentially adhere to larger blood vessels, in contrast to SLE pneumonia, in which they are deposited mainly in smaller vessels [67]. The described mechanisms of PAH-SLE emphasize the complex pathogenesis of the processes leading to pulmonary changes.

### 4.3. Pathogenesis of PAH in MCTD

Data on MCTD-PAH are scarce, due to the lower incidence of this disease compared with SLE and SSc. Moreover, the prevalence of PAH in MCTD is difficult to estimate because patients may be misdiagnosed with different overlap syndromes [36]. Contrary to SLE, patients with SSc-PAH and MCTD-PAH are characterized by more pronounced fibrosis, including fibrous thickening of the intima [66]. However, the exact pathological mechanism of PAH in MCTD remains unknown. Nevertheless, attempts are being made to define pathogenic pathways and identify possible risk factors. Among them, the role of antibodies in the development of MCTD-PAH is emphasized, such as anti-ribonucleoprotein-1 (anti-U1-RNP) [34], anti-β_2_-GPI IgG [34], and anti-anticardiolipin antibodies [68].

The role of anti-U1-RNP antibodies in the development of PAH-MCTD is ambiguous. Some authors emphasize that their presence is associated with better survival than in patients with U1-RNP negative [69]. In turn, the study by Hajas et al. shows that high levels of anti-U1-RNP antibodies may contribute to endothelial cell proliferation. The authors proved that patients with PAH-MCTD have consistently high levels of anti-U1-RNP antibodies (>30 U/mL) [32]. Similarly, the presence of elevated titers of anticardiolipin antibodies, anti-β2-glycoprotein I, and anti-endothelial cell antibodies increases the risk of mortality in MCTD regardless of the development of PAH [32].

The second cause of the development of MCTD-PAH is ILD, which occurs in 50–70% of patients with MCTD [70]. Among the predisposing factors, symptomatic heart disease is also included, which is caused by left ventricular diastolic dysfunction observed in one-third of patients [71]. Interestingly, MCTD patients with predominant SSc features have a higher prevalence of PAH than patients with typical MCTD and anti-anti-U1-RNP [34]. The described risk factors for PAH development in the course of MCTD require further studies in larger groups of patients.

### 4.4. Pathogenesis of PAH in Rheumatoid Arthritis

PAH secondary to RA is rare [35,72]. It is worth noting that PAH-RA can be caused by cardiac complications (ischemic heart disease and heart failure with preserved ejection fraction) [73] or pulmonary disorders (ILD) [74].

In RA, systemic inflammation is characterized by increased levels of circulating proinflammatory cytokines, which lead to endothelial dysfunction through increased generation of reactive oxygen species [75]. As a result of these processes, the bioavailability of nitric oxide (NO) is reduced [76]. Among the activators of nitrosative and oxidative stress, interleukin 6 and TNF-alpha play a key role. These cytokines contribute to increased myocardial stiffness and fibrosis, which ultimately leads to diastolic dysfunction due to impaired myocardial relaxation [77].

Therefore, IL-6 and TNF-alpha levels are used to stratify the risk of diastolic dysfunction [78]. Hence, RA patients with active disease and systemic inflammation have an increased risk of HFpEF [79]. Evidence of coexisting inflammation with fibrosis includes infiltration of transforming growth factor (TGF) β-producing leukocytes and increased collagen levels in the endomyocardium of patients with diastolic dysfunction [80]. In addition, biopsy studies confirm immune cell infiltration in cardiac tissue, which is associated with increased expression of proinflammatory cytokines and adhesion molecules [81].

### 4.5. PAH Pathogenesis in Sjögren Syndrome

The prevalence of PAH associated with primary Sjögren’s syndrome (pSS) is relatively high compared to the general population. In the Greek cohort, pulmonary hypertension is reported in about 20% of patients with pSS and is mainly mild [82] PAH in pSS may develop as a result of valvular heart disease (group 2), interstitial lung disease—ILD (group 3), or have an Idiopathic background (group 1) [83,84,85]. Thus, PAH in pSS may arise from interstitial fibrosis with increased pulmonary vascular resistance or direct vascular proliferation (resulting from remodeling of small pulmonary arteries), independently of the involvement of the lung parenchyma. Furthermore, chronic inflammation and immune-mediated endothelial damage contribute to the development of PAH and right ventricular overload [86]. In this process, autoimmune disorders with B cell stimulation predominate. Therefore, elevated parameters such as anti-Ro/SSA and anti-RNP antibodies, positive rheumatoid factor, and hypergammaglobulinemia may be markers for the development and progression of pSS-PAH [87]. Other data suggest that the risk of PAH is increased in patients with early-onset pSS and positive anti-SSB or anti-U1RNP antibodies [88].

Excessive endothelial cell proliferation, along with neoangiogenesis, can result in the formation of glomerular structures known as plexiform lesions, which are typical of PAH. Immune complexes and complement deposits are detected on the walls of pulmonary arteries, triggering chronic inflammation, endothelial dysfunction, and vascular remodeling [87]. This process is associated with altered synthesis of various endothelial vasoactive mediators, such as NO, endothelin-1, prostacyclin, and thromboxane [87,89]. Elevated levels of endothelin-1 cause pulmonary vascular hypertrophy and the structural remodeling typical of pulmonary hypertension [89,90]. Vasoconstriction and synthesized autoantibodies further enhance endothelial damage, promoting a prothrombotic and proinflammatory environment [91]. In addition, elevated proinflammatory and profibrotic mediators such as TNF-α and TGF-β induce vascular remodeling and increase vascular stiffness and resistance in both the pulmonary and systemic circulation [92].

Recent data show that the lung lesions are dominated by submucosal mononuclear cell infiltration, consisting mainly of CD4+ T cells. This suggests a similar proinflammatory process in the lungs as in the salivary glands, with epithelial cells playing a key role in the initiation, maintenance, and symptomatology of the disease [93]. Increased CCL5 expression and CD8 T cells are key regulators of immunopathological pathways in pSS [94]. Another PAH marker is the chemokine RANTES, which is an important chemoattractant for monocytes and T cells and can promote cell recruitment in the lungs of patients with severe PAH. Increased RANTES mRNA expression is associated with CD45+ inflammatory cell infiltrates in patients with severe PAH compared with healthy individuals [95].

### 4.6. Pathogenesis of PAH Invasculitis

PAH is a rare complication of TAK among various pulmonary pathologies. Pulmonary artery involvement in TAK is reported in 14.6% of patients [96]. The etiology of PAH-TAK is largely unknown; however, the disease is characterized by inflammatory granulomatous vasculitis of medium- and large-artery vessels. Inflammation includes mononuclear cell infiltration and formation of giant cell granulomas, which are associated with reactive fibrosis and increased ground substance in the intima. Inflammation also occurs around the vasa vasorum and at the medial-adventitial junction. As a result, transmural fibrous thickening of the arterial walls of large vessels is observed, causing multiple vascular occlusions. These vascular changes can lead to ischemic changes and pulmonary hypertension [44,97]. Wang et al. reported that right-sided pulmonary artery lesions are more common than left-sided changes in TAK patients with pulmonary hypertension and the most common finding is pulmonary artery occlusions (88.9%), followed by pulmonary artery stenosis and poststenotic dilation [98]. In contrast, the study by Mukoyama et al. reported that right-sided pulmonary artery involvement was the predominant finding, and similarly to Wang et al., confirmed that pulmonary artery obstruction was the most common finding (in 54.2% of patients) [96].

In the case of medium-vessel vasculitis, which includes Behçet’s disease, pulmonary hypertension may be caused by left heart disease (group II PH), pulmonary artery involvement (group IV), or chronic obstructive pulmonary disease (group III) [45]. A unique feature of Behçet’s disease is venular involvement and the formation of pulmonary and arterial aneurysms, which is the main cause of death in this group of patients [99,100]. This process involves activated T helper type 1 (Th1) cells, leading to an increase in the number of circulating T lymphocytes. Excessive macrophage activation, neutrophil chemotaxis, and phagocytosis are also observed [47]. Circulating immune complexes play a role in causing the characteristic vascular neutrophilic reaction. In addition, antibodies against endothelial cells are synthesized, which participate in endothelial cell dysfunction [101]. Pulmonary embolism is very rare and is caused by in situ thrombosis rather than peripheral thromboembolism [102].

In ANCA-associated small vessel vasculitis (AAV), pulmonary involvement with granuloma formation is typical of GPA. Lung lesions are less common in MPA compared with GPA, but when capillaritis develops, it causes severe alveolar hemorrhage. Pulmonary fibrosis may also develop in MPA, which rarely leads to PAH. Patients are at increased risk of hypertension and coronary artery disease, which cause left heart disease, one of the risk factors for PAH-AAV [46].

## 5. PAH-CTD Treatment

**Figure 2:**
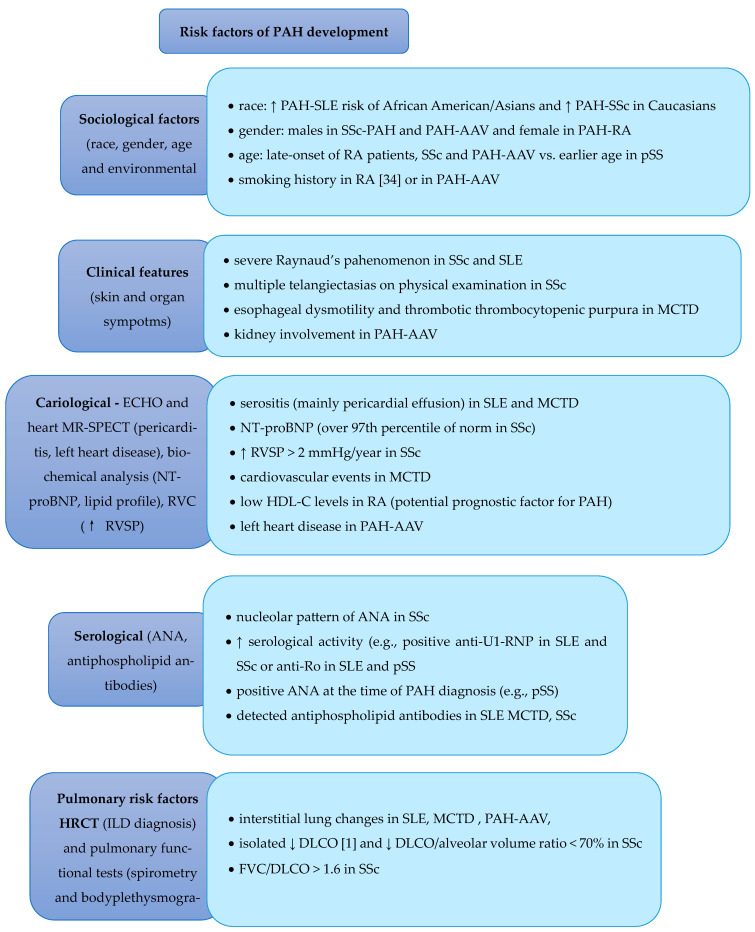
Risk Factors of PAH Development and Corresponding Treatment [1,2,3,4,5,6,7,8,9,10,12,16,21,22,23,25,26,28,31,35,37,38,39,45,46], ↑—increase, ↓—decrease.

A comprehensive therapeutic strategy and multidisciplinary care are essential in managing patients with PAH. In addition to specific pharmacological treatments for PAH, general management principles and monitoring procedures are integral to optimal patient care. Figure 2 provides a detailed overview of the factors influencing the choice of the appropriate treatment options.

### 5.1. Specific Therapy

Specific medications for PAH should be prescribed to patients with PAH-CTD following the same therapeutic algorithm used for IPAH. The primary classes of drugs utilized in the treatment include calcium channel blockers for those with a positive vasodilatation test, endothelin receptor antagonists, phosphodiesterase type 5 inhibitors, and guanylate cyclase stimulators, as well as prostacyclin analogs and prostacyclin receptor agonists.

### 5.2. Calcium Channel Blockers

Calcium channel blockers (CCBs) are used in patients who have had a positive response to an acute vasoreactivity test. Inhaled nitric oxide (NO), inhaled iloprost, or intravenous epoprostenol are recommended as vasodilators in the vasoreactivity test [103]. A positive response is defined as a reduction in mean pulmonary artery pressure (mPAP) of ≥10 mm Hg to an absolute mPAP ≤ 40 mm Hg with an increase or no change in cardiac output [104]. The commonly used CCBs in PAH include nifedipine, diltiazem, and amlodipine [104]. Effective daily doses for PAH are relatively high and are achieved by gradually increasing the dose. Common adverse effects include hypotension and peripheral edema. Patients with a positive acute vasoreactivity test receiving CCBs should be closely monitored for safety and efficacy. Reassessment should be conducted 3–4 months after initiation of therapy, including right heart catheterization (RHC). For patients who have not undergone an acute vasoreactivity test or who have a negative response, CCB therapy is not recommended for PAH due to the risk of serious adverse effects. However, CCBs may be prescribed for other indications, such as systemic hypertension, Raynaud’s phenomenon, or arrhythmias, depending on individual patient needs and comorbidities [50].

### 5.3. Endothelin Receptor Antagonists

The main representatives of this class include ambrisentan, macitentan, and bosentan. Their pharmacological mechanism involves inhibiting endothelin receptors A and B. Binding of endothelin-1 to endothelin receptors type A and B on the smooth muscle cells of the pulmonary artery promotes vasoconstriction and cell proliferation. Endothelin receptor type B is primarily located on the endothelial cells of pulmonary vessels, causing vasodilation through increased production of prostacyclins and nitric oxide and elimination of endothelin-1 [105]. However, comparable efficacy has been shown for selective inhibition of endothelin receptor type A, and non-selective inhibition of endothelin receptors type A and B in PAH patients. Endothelin receptor antagonists should not be used in pregnant women due to their teratogenic effects [106].

### 5.4. Phosphodiesterase Type 5 Inhibitors and Guanylate Cyclase Stimulators

Key drugs in this category include sildenafil, tadalafil, and riociguat. Sildenafil and tadalafil, which are phosphodiesterase type 5 inhibitors (PDE5i), stimulate the NO-cGMP pathway by slowing the breakdown of cGMP. Riociguat, a guanylate cyclase stimulator, increases cGMP production through direct interaction with soluble guanylate cyclase stimulator (sGC), both in the presence and absence of endogenous nitric oxide [107].

### 5.5. Prostacyclin Analogs and Prostacyclin Receptor Agonists

In PAH patients, reduced expression of prostacyclin synthase in pulmonary arteries and decreased levels of prostacyclin metabolites in urine have been observed [108]. Prostacyclin analogs and prostacyclin receptor agonists strongly dilate blood vessels, inhibit platelet aggregation, and additionally exhibit cytoprotective and antiproliferative effects [109]. Epoprostenol is administered via continuous infusion using an infusion pump and a permanent tunneled central catheter, iloprost is given by inhalation, and treprostinil is available for subcutaneous, intravenous, inhaled, and oral administration. Selexipag is an orally available selective prostacyclin receptor agonist, chemically and pharmacologically distinct from endogenous prostacyclin.

### 5.6. Non-Specific Therapy

Certain aspects of PAH-CTD treatment may vary depending on the underlying CTD. Immunosuppressive therapy with glucocorticoids and cyclophosphamide may improve clinical outcomes in patients with PAH associated with SLE or mixed CTD [110]. However, steroids are not recommended for patients with PAH-SSc (they increase the risk of scleroderma renal crisis) [111]. Patients with SSc and other CTDs may have concurrent ILD and/or HFpEF, which should be considered when deciding to initiate PAH pharmacotherapy [41]. The risk-benefit ratio of long-term oral anticoagulant therapy in SSc patients is unfavorable due to the increased risk of bleeding. However, anticoagulation with vitamin K antagonists is recommended in PAH-CTD patients with a predisposition to thrombophilia (e.g., in the secondary antiphospholipid syndrome) [112]. Diuretics are useful for treating symptoms of congestion. Vaccination is particularly important in this group of patients to reduce the risk of infections, such as respiratory infections, which can exacerbate pulmonary hypertension and contribute to disease progression. Vaccines against influenza and pneumococcus are routinely recommended for patients with CTD and PAH, as these infections can significantly worsen clinical outcomes in this already high-risk population. Supervised physical exercise and oxygen therapy are beneficial when they provide documented symptomatic improvement and address correctable exercise-induced desaturation [113]. Additionally, correcting iron deficiencies, which are associated with deteriorating cardiac function, worsening of the underlying disease, and higher mortality risk, is essential [114].

## 6. Treatment Strategies for PAH-CTD Based on ESC 2022 Guidelines for the Diagnosis and Treatment of Pulmonary Hypertension

For risk stratification at the time of diagnosis, it is recommended to use a three-level model, incorporating as many variables as possible (Table 2a), with a particular focus on disease subtype, WHO functional class (WHO-FC), 6-min walk test (6MWT), BNP/NT-proBNP levels, and hemodynamic parameters. During the follow-up period, a four-level PAH risk assessment model is recommended (Table 2b) [15].

**Figure 3:**
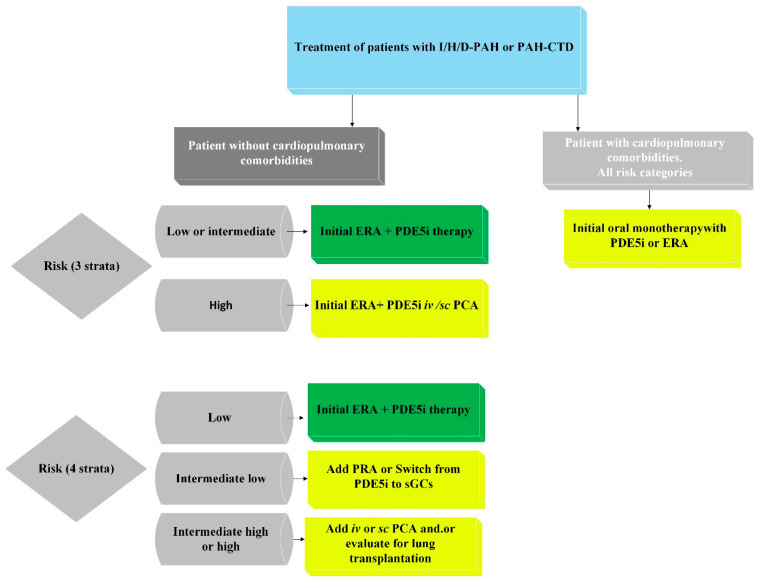
Proposed treatment regimen for PAH associated with connective tissue disease (PAH-CTD), based on ESC 2022 guidelines for the diagnosis and treatment of pulmonary hypertension and developed by the authors to summarize and illustrate key therapeutic steps.; ERA—endothelin receptor antagonist; I/H/D-PAH—idiopathic, heritable, or drug-associated pulmonary arterial hypertension; iv—intravenous; PAH-CTD—PAH associated with connective tissue disease; PCA—prostacyclin analog; PDE5i—phosphodiesterase 5 inhibitor; PRA—prostacyclin receptor agonist; sc—subcutaneous; sGCs—soluble guanylate cyclase stimulator.

Additional variables, such as imaging and right heart hemodynamic data, should be considered in patients with difficulties at the diagnostic step at the onset of PAH symptoms. However, at any stage of the disease, individual factors such as age, sex, disease subtype, comorbidities, and renal function must be taken into account. For patients identified as being at high risk of mortality, initial combination therapy, including PDE5 inhibitors, endothelin receptor antagonists (ERAs), and intravenous/subcutaneous prostacyclin analogs, should be considered. For patients at low or intermediate risk, initial combination therapy with PDE5 inhibitors and ERAs is recommended. If patients on combination therapy with ERAs and PDE5 inhibitors show intermediate-high or high risk of mortality, additional inclusion of intravenous/subcutaneous prostacyclin analogs and evaluation for lung transplantation (LTx) should be considered. If patients show an intermediate-low risk of mortality during combination therapy with ERAs and PDE5 inhibitors, the addition of selexipag is recommended, and switching from PDE5 inhibitors to riociguat is also advised. The treatment algorithm presented in Figure 3 is a schematic representation developed by the authors based on the current ESC guidelines and is intended to provide a practical summary adapted to patients with PAH associated with connective tissue diseases [15].

## 7. Conclusions

As many studies have described different classification criteria and methods used to diagnose PAH, the available literature data are limited and contradictory. However, PAH frequently develops in systemic sclerosis, SLE, and MCTD patients. Nevertheless, it can also develop in any CTD. Increased awareness and careful analysis of CTD patients with diagnosed pulmonary disorders may help identify subjects with PAH and thus enable immediate treatment. Moreover, in the group of idiopathic PAH patients, non-specific symptoms of CTD (e.g., systemic sclerosis without skin changes or Sjogren syndrome without sicca symptoms) may cause misdiagnoses of rheumatic diseases and thus loss of chance to use immunosuppressants and effective treatment. Therefore, in each patient with CTD who reported dyspnea or any other symptoms that suggested pulmonary problems, PAH risk should be immediately estimated. Conversely, patients initially presenting with PAH should be considered for potential CTD.

## Figures and Tables

**Table 1:** Risk factors of pulmonary arterial hypertension in connective tissue diseases.

Risk Factors of PAH in CTD	
Disease	Risk Factors
**Risk factors of SSc-PAH**	severe Raynaud’s phenomenon and severe fingertip ulcers [1] many telangiectasias on physical examination [2] race (African American) and gender (male)—according to the PHAROS [22,23]. longer disease duration and late-onset SSc [17,24] nucleolar pattern of ANA [8] antibodies: against ACA, Th/To, B2-GPI, U1-RNP + absence of anti-Scl 70 [8] isolated ↓ DLCO [1] ↓ DLCO/alveolar volume ratio < 70% + ↑ NT-proBNP > 97th percentile of normal [27] FVC/DLCO > 1.6 and ↑ RVSP > 2 mmHg/year [27]
**Risk factors of SLE-PAH**	serositis [4,9] (mainly pericardial effusion) [10,12] mild ILD [9,10] Raynaud’s phenomenon [4,12]—a marker of PAH severity [28] ↑ increased serological activity [5] positive aCL and anti-U1-RNP [4,10,12] positive lupus anticoagulant (LA), aβ2-GPI [7], anti-Ro, anti-La [6,10], and anti–Scl–70 detected in SLE patients [11] hyperuricemia—uric acid level ≥ 6.5 mg/dL [8] or UA ≥ 7 mg/dL [12] interstitial lung changes and aCL (immunoglobulin G class) [12] LA and APS [29]
**Risk factors of MCTD-PAH**	cardiovascular events, secondary APS [32] antibodies: anti-Sm [30], anti-aCL, anti-β2-GPI, anti-endothelial cell antibodies [32] ILD Thrombocytopenia Serositis (mainly pericarditis) esophageal dysmotility thrombotic thrombocytopenic purpura [32] absence of polyarthritis [30]
**Risk factors of RA-PAH**	exposure to environmental dust or cigarette smokers mainly in older RA at disease onset (~64.0 vs. 53.7 years) [36] lower baseline mPAP [36] positive correlation with disease duration and disease activity (*n* = 75 RA patients) [37] PAH correlates with RA duration and DAS disease activity score low C-HDL levels (potential prognostic factor for PAH in RA patients) [38,39] female gender [40]
**Risk factors of pSS-PAH**	earlier age and shorter duration of pSS [47] ↑ anti-SSA/Ro antibodies positive ANA at the time of PAH diagnosis [3]
**Risk factors of PAH in various vasculitis**	↑ pulmonary vessel wall stiffness Takayasu arteritis [44] pulmonary vascular changes in Behçet’s disease [45] risk factors of PAH-AAV: left heart disease chronic lung disease older age male gender smoking history kidney involvement [46]

ACA—antibody against centromere antibodies, aCL—anticardiolipin antibodies, APS—antiphospholipid syndrome, C-HDL—high-density lipoprotein cholesterol, DAS—disease activity score, DLCO—diffusing capacity of carbon monoxide, FVC—forced vital capacity, ILD—interstitial lung disease, LA—lupus anticoagulant, mPAP—mean pulmonary artery pressure; NT-proBNP—terminal pro-brain natriuretic peptide, PHAROS—the Pulmonary Hypertension Assessment and Recognition of Outcomes in Scleroderma Registry, RVSP—right ventricular systolic pressure, Th/To, B2-GPI—anti-beta2-glycoprotein, U1-RNP—U1-ribonucleoprotein, and the absence of anti-Scl 70 antibodies. ↑ -increase, ↓-decrease

**Table 2:** Three-step and four-step Risk Assessment Model for PAH-CTD. (a). Three-step Risk Assessment Model for PAH-CTD (stratification at the time of diagnosis). (b). Four-step Risk Assessment Model for PAH-CTD (stratification during the follow-up period).

(a)				
Determinants of Prognosis (Estimated 1-Year Mortality)		Low Risk (<5%)	Intermediate Risk (5–20%)	High Risk (>20%)
**Clinical observations and modifiable variables**				
Signs of right HF		Absent	Absent	Present
Progression of symptoms and clinical manifestations		No	Slow	Rapid
Syncope		No	Occasional syncope	Repeated syncope
WHO-FC		I, II	III	IV
6MWD		>440 m	165–440 m	<165 m
**CPET**				
Peak VO2		>15 mL/min/kg	11–15 mL/min/kg	<11 mL/min/kg
		>65% pred.	35–65% pred.	<35% pred.
VE/VCO		slope < 36	slope 36–44	slope > 44
**Biomarkers**				
BNP		<50 ng/L	50–800 ng/L	>800 ng/L
NT-proBNP		<300 ng/L	300–1100 ng/L	>1100 ng/L
**Echocardiography**				
RA area		<18 cm^2^	18–26 cm^2^	>26 cm^2^
TAPSE/sPAP		>0.32 mm/mmHg	0.19–0.32 mm/mmHg	<0.19 mm/mmHg
pericardial effusion		No	Minimal	Moderate or large
**cMRI**				
RVEF		>54%	37– 54%	<37%
SVI		>40 mL/m^2^	26–40 mL/m^2^	<26 mL/m^2^
RVESV		<42 mL/m^2^	42–54 mL/m^2^	>54 mL/m^2^
**Hemodynamics**				
RAP		<8 mmHg	8–14 mmHg	>14 mmHg
Cl		≥2.5 L/min/m^2^	2.0–2.4 L/min/m^2^	<2.0 L/min/m^2^
SVI		>38 mL/m^2^	31–38 mL/m^2^	<31 mL/m^2^
SvO_2_		>65%	60–65%	<60%
**(b)**				
**Determinants of Prognosis**	**Low Risk**	**Intermediate Risk**		**High Risk**
		**Low Risk**	**High Risk**	
Points assigned	1	2	3	4
WHO-FC	I or II	-	III	IV
6MWD	<440 m	320–440 m	165–319,	<165 m
BNP	<50	50–199	200–800	>800
NT-proBNP	<300 ng/L	300–649 ng/L	650–1100 ng/L	>1100 ng/L

Notably, 6MWD—6-min walking distance; BNP—brain natriuretic peptide; CI—cardiac index; cMRI—cardiac magnetic resonance imaging; CPET—cardiopulmonary exercise testing; HF—heart failure; NT-proBNP—N-terminal pro-brain natriuretic peptide; PAH—pulmonary arterial hypertension; pred., predicted; RA—right atrium; RAP—right atrial pressure; sPAP—systolic pulmonary arterial pressure; SvO_2_—mixed venous oxygen saturation; RVESVI—right ventricular end-systolic volume index; RVEF—right ventricular ejection fraction; SVI—stroke volume index; TAPSE—tricuspid annular plane systolic excursion; VE/VCO_2_—ventilatory equivalents for carbon dioxide; VO _2_—oxygen uptake; WHO-FC—World Health Organization functional class. Notably, Risk is calculated by dividing the sum of all grades by the number of variables and rounding to the next integer.

## Data Availability

This review represents original work and has not been duplicated or published in full or in part in any other publication or language. All data generated or analyzed during this study are included in the published article.

## Abbreviations

CTDs	connective tissue diseases,
MCTD	mixed connective tissue disease,
PAH	Pulmonary arterial hypertension,
pSS	primary Sjögren’s syndrome,
SLE	systemic lupus erythematosus,
TTE	transthoracic echocardiograph
